# Latent Gonorrhea in the Male as a Factor in Diseases of the Female Organs of Generation

**Published:** 1900-04

**Authors:** Lewis Wheat

**Affiliations:** Richmond, Va., Professor of Diseases of the Genito-Urinary Organs and Syphilis in the University College of Medicine.


### LATENT GONORRHEA IN THE MALE AS A FACTOR IN DISEASES OF THE FEMALE ORGANS OF GENERATION.*

By LEWIS WHEAT, M.D., RICHMOND, VA.,

Professor of Diseases of the Genito-Urinary Organs and Syphilis in the University College of Medicine.

That gonorrhea plays an important part in the diseases of the female organs of generation is now almost universally conceded. The extent to which it may remain latent in the male when appar-

* Read before the Richmond Academy of Medicine and Surgery, February 27, 1900.

ently cured, and the frequency with which the female is infected under such circumstances, is still, however, a matter of dispute. I believe it to be a source of frequent infection; and my object to-night in reading this paper is not so much to tell you something new as to refresh your memories and induce you to impress your patients with the necessity for more careful treatment of the disease.

There is no malady that has been more grossly exaggerated on the one hand and underestimated on the other than gonorrhea. The vast majority of the cases are, and remain, especially when properly treated, a simple local disease, readily cured in both male and female; while a few tax to the utmost our skill and patience in their treatment, and through systemic infection, often in spite of our best efforts, become a horrible disease, causing intense suffering and frequently ending in irremediable deformity or death. There is, too, an intermediate class, in whom the disease seems to lie ever dormant. It is, apparently, cured readily enough, but as readily breaks out again. I suppose that every one of you has some old stand-by, who is sure to turn up once in six months with a case of clap. It is this class in whom the disease seems to be latent, giving no evidence of its existence for months, or as long as the patient conducts himself properly. It is certain to break out, however, upon any debauch in venery or dissipation. There is another class, very much rarer: Those whose religious character is at stake and who have transgressed but once, and who, through shame and mortification, secretly treat themselves, or allow their disease to wear itself out; these are most dangerous from our present point of view. This class marry with the germs still lurking in the crypts and follicles of the urethral mucous membrane and the recesses of the prostate and vesicula seminales, their long presence having established a toleration. These are almost certain to infect their wives.

In 1887 I was sent for to see a young woman recently married, who had returned prematurely from what was to have been an extensive bridal tour, in consequence of a slight indisposition. Her trouble was apparently not very serious, but sufficient to render further travel irksome rather than a source of pleasure. At the time of her marriage she was in perfect health, and had never been seriously sick in her life. When I saw her, about six weeks after her marriage, she complained of no well-defined trouble. She had menstruated at her usual time, but had noticed nothing very unusual except that her menstrual flow was a little more profuse and accompanied by some pain and a slight discharge other than menstrual. It was not enough, however, to attract attention, and she had only sent for me at the solicitation of her husband, more on account of a sense of fatigue when she got out of bed in the morning, accompanied by slight nausea and headache, loss of appetite and a feeling of lassitude and indifference, than anything else. Her husband was a Y. M. C. A. man, prominent in his church, and had borne an unblemished character as a model young man from his youth up. It never entered my head to even suspect that he had ever had the clap. I told the patient that she was bilious and suffering from indigestion, and probably pregnant; gave her a prescription and left to call again in a day or two. When I next saw her she said she was a little better, but not well, and I gave her another prescription for indigestion, to earn my fee, and put her on a diet, still thinking that she was probably pregnant, which would account for some of her symptoms. When I next saw her she was menstruating, and suffering even more pain than at her former period. The flow, except being a little more profuse, was still apparently perfectly natural and free from any unnatural discharge. There was considerable suprapubic tenderness, and her sexual relations caused violent pain, lasting for some time after the completion of the act, and leaving her very much exhausted. This condition of affairs continued for some time without improvement, and I now attributed her trouble to a mild pelvic cellulitis caused by too frequent and too violent sexual relations. At her request, her old physician saw her with me and concurred in my diagnosis. We treated her with hot applications to the abdomen, copious vaginal irrigations of very hot water night and morning, and repeated small blisters over the ovarian region. Under this treatment she slowly improved and was able to be up and about. But she now suffered occasionally with horrible pelvic pain lasting a week or ten days, during which time it was necessary to keep her constantly under the influence of morphia and atropia. After a discharge of a tablespoonful of a mucilaginous fluid into the vagina, she would be comparatively free from pain, sometimes for several weeks at a time. This state of affairs continued, with some improvement, for nearly ten years, the intervals between her attacks of pain becoming longer and the pain shorter and less severe. Four years ago she became pregnant, and in time was delivered of a fine boy.

So dense was my ignorance and stupidity, I never suspected the cause of this woman's trouble until the meeting of the Southern Surgical and Gynecological Society in this city in November, 1891, four years after her marriage. During the discussion of Dr. Brown's paper on "Systemic Infection from Gonorrheal Poisoning," my eyes were suddenly opened to the truth. Her husband acknowledged that he had fallen from grace and contracted gonorrhea, which he had treated himself with injections. After lasting a long time the discharge stopped, and he believed himself well. You must remember that at that time none of us knew much about gonorrhea in women. And how many of you, unprompted, will suspect the existence of gonorrhea in a bride of six weeks whose husband is regarded as immaculate? My observations have led me to believe that infection from such a source rarely gives rise to more marked symptoms than those in the case just related; and these are often unaccompanied at any stage by pus or adhesions of any moment.

I assisted Dr. W. H. Parker, some time ago, in removing the tubes and ovaries in a similar case, in which these organs showed every sign of disease except the presence of pus and adhesions.

One reason for writing this article is to call attention to the fact that gonorrhea in the female is rarely discovered until irretrievable damage has been done; and to suggest that, if we accept the teachings of Noeggerath, Tait, Price and other eminent gynecologists, as we must do at least in part as confirmed by our own experience, it is our duty to impress upon our patients the serious consequences that may follow an attack of gonorrhea, and the necessity for early and thorough treatment; and also when they marry the necessity, if they wish to escape the serious consequences that so often follow gonorrhea in women, for its early recognition and prompt treatment if their wives are infected. This is the more important, as the disease in women rarely presents the clinical features we would expect, and is seldom recognized as venereal in its origin. How many cases of gonorrhea in women have any of us been called upon to treat as such? I can only recall three, outside of children who were infected by their nurses. Lydston, of Chicago, in his valuable work just issued, says: "If the more advanced views be correct, the correlations of gonorrhea and gleet in the male are too frequently regarded as simple and non-venereal in the female. It is a striking clinical fact that gonorrheal inflammation in the female rarely presents conditions physically analogous to those observed in the male. Vaginitis of venereal origin is exceptional, and urethritis, the only real analogue, is excessively rare in women, and, moreover, does not always occur when virulent vaginitis exists." While the disease, as we have seen, may be as mild or even less troublesome in women than in men, the loss of her sexual organs and untold agony can often only be prevented by the early recognition of the disease and its prompt treatment. If recognized sufficiently early, I believe that gonorrhea is more certainly and easily cured in women than in men. "The researches of modern operative gynecologists are developing most astonishing facts. The more carefully we study pelvic diseases in women, the narrower their etiologic field becomes, the more frequently they are found to be dependent upon gonorrhea. Thus, when freed from pathologic and anatomic errors, pelvic inflammations are found to be dependent, in the majority of cases, if not all, upon tubal disease, and tubal disease is unquestionably almost always due to gonorrhea and its congeners or derivatives." (Lydston.)

Noeggerath, applying the dictum of Ricord that in 1,000 men 800 have had gonorrhea, says: "I do not believe that I exaggerate when I say that gonorrhea in 90 per cent. of cases remains uncured. Of every hundred women who have married men formerly affected by gonorrhea, hardly ten remain well; the others are afflicted by some of the ailments that I have attempted to describe." I know from my own experience that this is an exaggeration. The young men of Paris, New York and Chicago may be so unfortunate, but it is not the case in Virginia. I know that I have treated many patients who have married, and are the fathers of many children, without infecting their wives. And I believe that when treatment is instituted sufficiently early, and properly and thoroughly carried out, clap is as completely eradicated as any other disease. It is the pernicious custom of treating the disease as a slight ailment of no consequence, and the fact that almost every drug store in the country is a clap shop, that produces the evil. I recognize the fact, however, that while the gynecologist is liable to error on his side, I am liable to err on my side. He sees the infected wives, and I see the men only, and in many instances lose sight of them after marriage. Yet when I did a general practice I treated many cases of gonorrhea and afterwards delivered their wives, and I am sure that a majority of them have never had any trouble that could by any possibility be attributed to gonorrhea. While the case I have detailed is one of many, all are by no means so fortunate. The literature of the last ten years teems with the reports of cases in which infection has been followed by metritis, endometritis, salpingitis, pyosalpinx, ovaritis, cystitis, ureteritis, pyelitis, peritonitis, acute pyemia, and death. I shall only occupy your time with one more case in my own experience which shows how rapidly general systemic infection may follow the disease when acquired from a man who thought that he was cured, and in whose disease the gonococci played but a very small part. Some time ago a young man whom I had apparently cured was married, and it is only fair to say, against my advice. When he came to me he was suffering from one of the most violent attacks of gonorrheal urethritis that I have ever seen. He had contracted the disease from a very handsome woman with whom he had spent the night about one week before seeing me. Two days before he had noticed a slight discharge, which had rapidly increased, and which was then pouring from a swollen and pouting meatus. The whole penis was swollen and excessively tender; the veins stood out like cords, and the lymphatics could be readily seen, enlarged, painful and excessively tender along the dorsum of the penis. Micturition was already so painful that an attempt to pass water caused great beads of perspiration to stand out upon his forehead; and that night and many nights and days thereafter it had to be drawn with a catheter, which caused such horrible agony that I was compelled to use chloroform. I put him on what was then my routine prescription: Santal-Midy and bicarbonate of soda, with sulphate of soda to keep his bowels open. Erections were almost constant and a source of agony. Sleep was impossible except under large doses of morphia and atropia. A hip-bath, as hot as could be borne, gave him some relief, and was frequently repeated. I also directed him to keep his penis immersed in a solution of chloral hydrate (five grains to the ounce of water), and when not so immersed or in the hip-bath, surrounded by absorbent cotton kept wet with the solution. In this way he passed his days longing for night, and his nights longing for day. His temperature ran from 100° to 101° F. Three days later it suddenly reached 104°—an almost certain indication of abscess in the prostate. Such it proved, rupturing a few days later while passing the catheter. Microscopic examination of the discharge showed gonococci in abundance and huge colonies of streptococci. After much suffering he recovered, with, of course, a much damaged urethra. He left Richmond shortly after, to accept an important position in another city. About eighteen months later he wrote to me that he was to be married in a few weeks. I replied, warning him that he could only do so with great risk of infecting his wife, and received the usual reply—that it was too late. Two weeks after marriage his wife was taken violently ill with so-called rheumatism. One joint after another became inflamed, followed by suppuration and ankylosis. More than six months passed in horrible suffering, when she was able to be moved and was sent to the Hot Springs. Since then I have heard little from either of them, except that she continues in wretched health and that her doctors advise an operation, the nature of which I do not know.

In the past twelve months I have seen three cases of suppurating appendicitis in young men suffering with acute gonorrhea. I very foolishly failed to have the pus examined, and so cannot say whether it was due to gonorrhea or not. From my own experience in systemic infection from gonorrhea, and the many reported cases, I believe it to be extremely probable. In such cases as the last related by me, no treatment can prevent their being dangerous as foci of infection for years, and I believe as long as they live. But an experience rather larger than usual leads me to believe that if physicians and patients would realize the serious nature of the disease and institute proper treatment at once, the disease could be eradicated and prevented from becoming latent. When, however, it is allowed to run on with improper treatment or none at all, for weeks or even months, Dr. Price is justified in saying that it never gets well and remains as a source of infection as long as the sufferer lives.

